# Do Paralympic athletes suffer from brittle bones? Prevalence and risk factors of low bone mineral density in Paralympic athletes

**DOI:** 10.1016/j.bonr.2024.101767

**Published:** 2024-04-18

**Authors:** Vera C.R. Weijer, Jan-Willem van Dijk, Lotte van Dam, Linn Risvang, Judith Bons, Truls Raastad, Luc J.C. van Loon, Kristin L. Jonvik

**Affiliations:** a School of Sport and Exercise, HAN University of Applied Sciences, Nijmegen, the Netherlands; b Department of Human Biology, NUTRIM, Maastricht University Medical Centre+, the Netherlands; c Department of Physical Performance, Norwegian School of Sport Sciences, Oslo, Norway; d Central Diagnostic Laboratory, Maastricht University Medical Centre+, Maastricht, the Netherlands

**Keywords:** Bone mineral density, Paralympics, Disability, Wheelchair, Energy availability, Osteoporosis

## Abstract

**Background:**

Bone health may be a concern in Paralympic athletes, given the presence of multiple risk factors predisposing these athletes to low bone mineral density (BMD). Objective: We aimed to assess the prevalence of low BMD among Paralympic athletes participating in various sport disciplines, and to identify potential risk factors for low BMD.

**Methods:**

Seventy Paralympic athletes, of whom 51 % were wheelchair-dependent, were included in this cross-sectional study. BMD of the whole-body, lumbar spine, total hip, and femoral neck were assessed by dual-energy x-ray absorptiometry. Comparisons between groups were conducted by one-way ANOVA, and regression analyses were conducted to identify potential risk factors for low BMD.

**Results:**

The prevalence of low BMD (*Z*-score < −1.0) was highest at femoral neck (34 %), followed by total hip (31 %), whole-body (21 %), and lumbar spine (18 %). Wheelchair-dependent athletes had significantly lower BMD Z-scores compared to the non-wheelchair-dependent athletes at whole-body level (−0.5 ± 1.4 vs 0.2 ± 1.3; *P* = 0.04), total hip (−1.1 ± 1.2 vs 0.0 ± 1.1; *P* < 0.01), and femoral neck (−1.0 ± 1.3 vs −0.1 ± 1.2; *P* < 0.01). At the lumbar spine, low BMD was completely absent in wheelchair basketball and tennis players. Regression analyses identified body mass, wheelchair dependence, and type of sport, as the main risk factors for low BMD.

**Conclusions:**

In this cohort of Paralympic athletes, low BMD is mainly present at the hip, and to a lesser extent at the whole-body and lumbar spine. The most prominent risk factors for low BMD in Paralympic athletes are related to mechanical loading patterns, including wheelchair use, the type of sport, and body mass.

## 1 Introduction

Physical activity generally has a positive impact on bone mineral density (BMD) (Chilibeck et al., 1995). In particular, high impact sports (e.g. rugby, running, weight lifting) are associated with a high BMD (Van Langendonck et al., 2003). In contrast, athletes of aesthetic or endurance sports are at risk for a low BMD, especially when the sport only involves low impact forces (e.g. cycling, swimming) (Gomez-Bruton et al., 2016; Nichols et al., 2003; Hilkens et al., 2023). Such a low BMD may predispose athletes to (stress)fractures as well as early osteoporosis (Goolsby and Boniquit, 2017). Therefore, acquiring a high peak bone mass early in life can prevent health issues both during and after the athletes' career.

While substantial knowledge on BMD is available in non-disabled athletes, such knowledge is currently limited for Paralympic athletes. Two relatively small studies indicated that ∼45 to 50 % of the Paralympic had low BMD (Z score ≤ 1) in at least one skeletal site (Cavedon et al., 2021; Koivisto-Mørk et al., 2023), although the prevalence of low BMD seemed substantially lower at individual skeletal sites (Koivisto-Mørk et al., 2023). In non-athletic adults with disabilities, the prevalence of low BMD is high. For example, Smith et al. *(*Smith et al., *2009**)* found that >50 % of this population has low BMD in at least one skeletal site. Possible risk factors for low BMD in this population are a reduced ambulatory status and a longer duration of the disability (Smith et al., 2009). Indeed, athletes with a spinal cord injury, who are predominantly wheelchair-dependent, had a significantly lower BMD both in the legs and at a whole-body level compared to non-disabled athletes (Miyahara et al., 2008). This finding in wheelchair-dependent athletes is likely attributed to the limited mechanical loading of the lower body, resulting in a lack of osteogenic stimulation (Robling et al., 2006). Aberrant mechanical loading can also be present in athletes with prostheses or abnormal gait patterns (e.g., cerebral paresis athletes), thereby affecting bone remodeling. Besides mechanical loading, insufficient dietary intakes can negatively affect BMD. In particular, low energy availability, which is defined as having insufficient energy to support the normal body functions, because of a mismatch between energy intake and exercise energy expenditure (Loucks et al., 2011), has a negative effect on BMD (Mountjoy et al., 2018). Based on hormonal markers of low energy availability, like low estradiol, insulin like growth factor 1, or triiodothyronine (T3), Paralympic athletes seem to be at risk for low energy availability (Pritchett et al., 2021) and, therefore, low BMD as well (Blauwet et al., 2017). Another risk to bone health in Paralympic athletes may be the use of certain medications such as anticonvulsants, corticosteroids, or thyroid hormones to combat the symptoms or comorbidities associated with their disability (Lee et al., 2010; Kim, 2010; Ribot et al., 1990). Despite the apparent increased risk for low BMD in Paralympic athletes, the prevalence and associated risk factors have yet to be established.

Regardless of the causes, it has been speculated that low BMD may predispose Paralympic athletes to traumatic fractures, even after minor falls or collisions (Webborn and Van de Vliet, 2012). This can be attributed to the increased fall risk due to reduced coordination (e.g. cerebral palsy), loss of proprioception (e.g. running on protheses), or unforeseen obstacles (e.g. visual impairment) (Webborn and Van de Vliet, 2012). Athletes with spina bifida or spinal cord injury may lack the sensation of a fracture in the lower body (Van de Vliet and Wilkinson, 2015), which can lead to a late diagnosis and treatment, thereby impeding the healing process and potentially necessitating surgical intervention and resulting in long-term impairment (Elam et al., 2023). Furthermore, fractures could hinder effective training and competitive participation, and even compromise athletes' independence due to limitations in performing daily activities (Vanlandewijck and Thompson, 2011). Besides traumatic fractures, low BMD may also predispose athletes to stress fractures. For example, among US Paralympic athletes the prevalence of sports related stress fractures, after the onset of disability is 9.2 % (Brook et al., 2019). Before defining effective prevention and treatment strategies to combat low BMD in Paralympic athletes, it remains to be determined which Paralympic athletes are at risk and what risk factors contribute to the development of low BMD in this population.

The current study evaluated BMD at different skeletal sites within a substantial cohort of Paralympic athletes with various disabilities across multiple sports. Additionally, this study investigated potential risk factors associated with low BMD in this population.

## 2 Methods

### 2.1 Study design

In this cross-sectional study, the BMD, body composition, training load, and blood markers of energy availability and bone status were assessed in Paralympic athletes (*n* = 70). This study was part of a larger collaborative study on the nutritional requirements of Paralympic athletes. Data were collected between September 2020 and September 2022 at the (mobile) sport and research center of HAN University of Applied Sciences (Nijmegen, The Netherlands) and at the department of Physical Performance at Norwegian School of Sport Sciences (Oslo, Norway). The study was approved by the Medical Ethical Committee Zuyd (NL72682.096.20) in the Netherlands and the Regional Committee for Medical and Health Research Ethics (REK 102284) in Norway.

### 2.2 Participants

Paralympic athletes competing in Para cycling (*n* = 17), wheelchair tennis (*n* = 9), wheelchair basketball (*n* = 18), Para alpine skiing (*n* *=* 8), Para Nordic skiing (*n* = 7) and other sports (*n* = 11; including Para swimming (*n* *=* 4), wheelchair rugby (*n* = 2), Para orienteering (*n* = 1), Para badminton (*n* = 1), Para rifle shooting (*n* = 1), Para snowboarding (*n* *=* *1*), and boccia (*n* = 1)), were recruited via the national coaches of the Dutch and Norwegian Olympic/Paralympic sport federations. In this exploratory study on the prevalence of low BMD among Paralympic athletes from the Netherlands and Norway, invitations were extended to all respective Paralympic teams. Without conducting a specific sample size calculation, we aimed to include any athlete from these teams willing to participate. Inclusion criteria were elite or talent level Paralympic athletes involved in a national sport program, aged 16 to 50 y. Multiple disabilities were included and categorized as spinal cord disorder, neurological impairment, amputation/dysmelia, visual/hearing impairment, and other disabilities. Participants were classified as ‘wheelchair dependent’ if >50 % of their ambulatory activities outside of sports were performed in a wheelchair. Exclusion criteria were the presence of serious injuries that restricted regular training, and pregnancy or lactation. All participants were informed about the nature and potential risks of the study and signed an informed consent according to code of ethics of the Declaration of Helsinki (World Medical Association, 2013).

### 2.3 Study procedures

Participants arrived at our (mobile) laboratories in the morning (between 7:00 and 10:00 h) in an overnight fasted state. Before the dual energy x-ray absorptiometry (DXA) scans, body mass was determined with a digital scale (Seca 770 and Seca 887, Hamburg, Germany, for the Netherlands and Norway respectively) and stature was determined with a mobile stadiometer (Seca 213i and Seca 437, Hamburg, Germany, for the Netherlands and Norway respectively). When the participants were unable to stand up straight, stature was measured from head to heel in supine position. Furthermore, a fasted venous blood sample was drawn, and an interview with specific questions about the sport and disability of the Paralympic athletes was conducted.

### 2.4 Measurements

#### 2.4.1 Bone mineral density and body composition

Three DXA systems were used, two in the Netherlands (Horizon and Discovery A, Hologic, MA, USA) and one in Norway (Lunar iDXA, GE Healthcare, Madison, USA). Whole-body BMD (g/cm^2^), lumbar spine (L1-L4) and left proximal femur (femoral neck and total hip) were determined by DXA procedures as recommended by the National Health and Nutrition Examination Survey (NHANES) (Control CfD, Prevention, 2013), using the system's software package (Hologic Horizon: Apex version 5.6.0.5, Hologic Discovery A: Apex version 4.5.3, Lunar iDXA: enCORE version 18). If a major implant was placed in the lumbar spine, the lumbar spine and whole-body scan were excluded. Minor metal implants, for example in the collarbone and wrist area, were omitted with the DXA software and the scans were included in analyses. Furthermore, in case the left hip was disformed because of the disability, hindering proper analysis (e.g., the neck box could not be placed at the femoral neck), the right hip was scanned and analyzed. If the same problem was encountered at the right hip, the hip scan was excluded for the femoral neck, but not for the total hip. Similarly, when the lumbar spine had severe scoliosis where the vertebrae could not be distinguished from each other, the lumbar spine scan was excluded. In addition to absolute BMD values, standardized BMD values were calculated for the lumbar spine, total hip and femoral neck according to the equations by Hui et al. *(*Hui et al., *1997**)* (for the lumbar spine) and by Lu et al. (Lu et al., 2001) (for the total hip and femoral neck), to account for potential discrepancies between the different DXA manufacturers. Furthermore, BMD values were standardized to *Z*-scores using age, ethnicity, and sex normative reference values of the respective DXA systems. The BMD Z-scores were used to assess the prevalence of low BMD (Z-score ≤ −1.0) and osteoporosis (Z-score ≤ −2.0), based on the ACSM classifications for athletes (ACSM, 2007). In addition to BMD, measurements of whole-body and regional body composition were performed, according to recommended standardization procedures (Nana et al., 2015), using the classic calibration algorithm (without NHANES correction). The DXA systems were calibrated according to manufacturer's instructions, in the morning before measurements took place.

#### 2.4.2 Blood markers

Fasted venous blood samples were taken from the antecubital vein. The blood samples were allowed to clot for ∼30 min and centrifuged at 1000g for 15 min. Subsequently, aliquots of serum were stored at −80 °C, until further analysis. Serum samples were analyzed by the Central Diagnostic Laboratory at the Maastricht University Medical Centre (The Netherlands) for T3, as marker of energy availability, and vitamin D (25-(OH)D), procollagen 1 intact n-terminal propeptide (P1NP) and C-terminal telopeptide 1 (CTX—I) as markers of bone status. Total T3 was determined with a chemiluminescent immunoassay (Immulite XPi instrument, Siemens Healthcare Diagnostics). Intact P1NP, CTX—I, and 25(OH)D were measured using chemiluminescence immunometric assays on the IDS-iSYS instrument (Immunodiagnostic Systems Holdings, PLC, Tyne and Wear, UK). Reliability (CV) values for between-day measurements were ≤ 9 % (CTX—I), ≤5 % (P1NP), ≤10 % (T3), and ≤ 9 % (25(OH)D). Low T3 was defined as <0.8 nmol/L and low vitamin D was defined as <50 nmol/L. Low P1NP, or CTX-I were based on reference values according to age and gender (Supplemental Table 1).

#### 2.4.3 Exercise training

All participants were asked to keep a training log with information on the timing, type and duration of all training sessions or competitions during a 14-day period. Some participants already tracked such data in a digital app, while the other participants were provided a written training log to record training data. Exercise duration was extracted from all exercise training logs and mean daily exercise duration served as a universal exercise training metric across sports.

#### 2.4.4 Data-analysis

All data were checked for normality with the Kolmogorov-Smirnov test. Normally distributed data are presented as means±SD, while non-normally distributed data are presented as median (Q1-Q3). Differences between wheelchair-dependent and independent athletes were analyzed with an unpaired *t*-test for normally distributed data or a Mann-Whitney *U* test when the data was non-normally distributed. Differences between sports were analyzed with a one-way ANOVA with Bonferroni correction for normally distributed data or a Kruskal-Wallis test when the data was non-normally distributed. Correlations were analyzed with either Pearson's correlation coefficient (normally distributed data) or Spearman's correlation coefficient (non-normally distributed data). Furthermore, multiple regression analyses with backward selection were conducted, with BMD *Z*-scores at different measurement sites (whole-body, lumbar spine, total hip, and femoral neck) as dependent variables. Potential risk factors for low BMD in Paralympic athletes were used as independent variables, including, body mass, years since disability onset, wheelchair dependence, vitamin D status, T3 (as a marker of energy availability), and type of sport (with the ‘other sports’ category being used as reference category). All statistical analyses were conducted with SPSS 27.0 (IBM Corp., Armonk, NY), with statistical significance set at *P* < 0.05.

## 3 Results

### 3.1 Participants

A total of 70 male (*n* = 34) and female (*n* = 36) Paralympic athletes were included (Table 1). Together, these athletes won a total of 11 gold, 6 silver, and 8 bronze medals during the last two Paralympic games (Tokyo 2020 and Beijng 2022, respectively). Athletes were classified as either having a spinal cord disorder (including spina bifida, traumatic and non-traumatic spinal cord injury; *n* *=* 21), neurological disorder (including cerebral palsy and other neurological disorders; *n* = 10), dysmelic or amputation (*n* = 18), a visual or hearing impairment (*n* = 7) or ‘other disabilities’ (*n* = 14). Other disabilities included nerve damage in the lower extremities, complex regional pain syndrome, intellectual impairments, spondylitis, connective tissue disease, muscular dystrophy, hip dysplasia, Perthes disease and arthrogryposis. Of all athletes 51 % were classified as wheelchair-dependent. Some athletes used medication that may interfere with bone metabolism, i.e., corticosteroids (*n* = 2), anticonvulsant (*n* = 1), and thyroid hormone (*n* = 1) during the study period, and a corticosteroid injection in the last six months before the study (*n* = 1). As shown in Table 1, no statistical differences were found between sports for any of the participant characteristics, except for training duration. None of the athletes exhibited a T3 concentration outside of the reference range (0.8 to 2.6 nmol/L). Eleven participants (17 %) had suboptimal levels of vitamin D (<50 nmol/L), with one participant having a severe vitamin D deficiency (<30 nmol/L). As shown in Table 2, wheelchair-dependent athletes had lower body height, body mass, lean body mass and serum P1NP concentrations, and higher age compared with non-wheelchair-dependent athletes.

**Table 1:** Participants' characteristics.

Sport	Total (*n* = 70)	Para cycling (*n* = 17)	Wheelchair tennis (*n* = 9)	Wheelchair basketball (*n* = 18)	Para alpine skiing (*n* = 8)	Para Nordic skiing (*n* = 7)	Other (*n* = 11)
Disabilities (*n*)							
Spinal cord injury	21	2	3	9	3	2	2
Neurological disorder	10	5	0	0	0	2	3
Dysmelic/amputation	18	5	5	3	3	1	1
Visual/hearing impaired	7	2	0	0	2	1	2
Other	14	3	1	6	0	1	3
Wheelchair dependent (no/yes)	34/36	12/5	2/7	7/11	3/5	4/3	6/5
Acquired at birth (no/yes)	34/36	8/9	3/6	14/4	4/4	3/4	2/9
Time since disability (years)	21 ± 11	22 ± 13	26 ± 9	17 ± 8	20 ± 7	21 ± 12	23 ± 11
Sex, male/female (*n*)	34/36	9/8	5/4	5/13	4/4	4/3	7/4
Age (years)*	25 (21−33)	26 (23−32)	27 (22−33)	26 (21−30)	28 (22–41)	23 (19–34)	21 (18–40)
Body height (cm)*	169 (160.4–180.0)	169.7 (164.0–185.8)	168.0 (140.9–176.5)	170.0 (153.1–179.3)	168.7 (129.1–174.8)	165.0 (151.1–180.0)	169.9 (160.0–180.0)
Body mass (kg)*	64.7 (55.5–72.5)	70.6 (55.7–73.7)	63.1 (53.4–68.2)	61.9 (56.6–78.6)	64.6 (61.4–71.9)	59.2 (51.0–72.5)	65.4 (55.3–69.5)
Lean body mass (kg)	47.4 ± 10.7	50.2 ± 12.0	46.5 ± 8.0	46.3 ± 11.2	46.6 ± 6.3	44.8 ± 10.4	47.9 ± 13.7
Training duration (h/week)	11.5 ± 4.8	12.4 ± 4.1	15.2 ± 5.6	8.5 ± 1.3^a^	7.7 ± 1.1^a^	13.8 ± 3.3	9.3 ± 5.9
T3 (nmol/L)*	1.39 (1.28–1.66)	1.27 (1.12–1.53)	1.42 (1.30–1.53)	1.36 (1.22–1.63)	1.53 (1.17–1.73)	1.37 (1.31–1.39)	1.67 (1.35–1.89)
Vitamin D25 (nmol/L)	67.2 ± 19.5	78.8 ± 20.2	62.4 ± 12.8	63.4 ± 16.6	73.1 ± 20.8	62.9 ± 17.0	58.4 ± 23.0
P1NP (ng/mL)	82.2 ± 35.3	84.9 ± 27.6	71.0 ± 33.8	70.8 ± 37.4	90.7 ± 34.4	74.0 ± 21.8	105.4 ± 43.8
CTX (ng/mL)	0.47 ± 0.31	0.56 ± 0.38	0.43 ± 0.25	0.35 ± 0.24	0.47 ± 0.33	N.A.	0.72 ± 0.31

Frequencies are presented as number of cases (*n*). Normally distributed data are presented as mean ± SD, while non-normally distributed data (*) are presented as median (Q1-Q3). No values for CTX for the Norwegian athletes were reported due to a technical error. The sports included in other sports: para swimming (*n* *=* 4), wheelchair rugby (*n* = 2), para orienteering (*n* = 1), para badminton (*n* = 1), para rifle shooting (*n* = 1), para snowboarding (*n* *=* *1*), boccia (*n* = 1) and wheelchair racing (*n* = 1). T3 = triiodothyronine; P1NP = procollagen 1 intact n-terminal propeptide; CTX = C-terminal telopeptide 1; N.A. = not applicable. ^a^ = significantly different from wheelchair tennis *P* < 0.05.

**Table 2:** Characteristics and bone mineral density of non-wheelchair-dependent and wheelchair-dependent Paralympic athletes.

	Total	Non-wheelchair-dependent	Wheelchair-dependent	*P*-value
Characteristics				
Age (years)*	25 (21–33)	23 (20–28)	30 (23–40)	**<0.01**
Body height (cm)*	169.0 (160.4–180.0)	175.0 (168.1–180.9)	164.0 (138.0–170.0)	**<0.01**
Body mass (kg)*	64.7 (55.5–72.5)	70.9 (61.3–77.7)	61.4 (52.8–67.3)	**<0.01**
Lean body mass (kg)	47.4 ± 10.7	52.9 ± 11.1	42.3 ± 7.4	**<0.01**
T3 (nmol/L)*	1.39 (1.28–1.66)	1.37 (1.27–1.63)	1.40 (1.30–1.68)	0.73
Vitamin D25 (nmol/L)	67.2 ± 19.5	66.8 ± 20.2	67.6 ± 19.2	0.87
P1NP (ng/mL)	82.2 ± 35.3	91.7 ± 34.9	73.7 ± 33.9	**0.04**
CTX (ng/mL)	0.47 ± 0.31	0.55 ± 0.31	0.41 ± 0.30	0.12
Bone mineral density (g/cm^2^)				
Whole-body	1.134 ± 0.122	1.168 ± 0.122	1.097 ± 0.113	**0.02**
Lumbar spine	1.083 ± 0.158	1.106 ± 0.142	1.053 ± 0.174	0.20
Total hip	0.916 ± 0.187	0.997 ± 0.164	0.835 ± 0.175	**<0.01**
Femoral neck	0.845 ± 0.199	0.913 ± 0.199	0.770 ± 0.174	**<0.01**
Bone mineral density *Z*-score				
Whole-body	−0.1 ± 1.4	0.2 ± 1.3	−0.5 ± 1.4	**0.04**
Lumbar spine	0.0 ± 1.3	0.2 ± 1.0	−0.1 ± 1.6	0.34
Total hip	−0.5 ± 1.3	0.0 ± 1.1	−1.1 ± 1.2	**<0.01**
Femoral neck	−0.5 ± 1.3	−0.1 ± 1.2	−1.0 ± 1.3	**<0.01**
Prevalence of low bone mineral density (*n*_*c*_/*n*_*t*_ (%))				
Whole-body	13/62 (21)	4/32 (13)	9/30 (30)	0.09
Lumbar spine	11/60 (18)	3/33 (9)	8/27 (30)	**0.04**
Total hip	21/67 (31)	3/34 (9)	18/33 (55)	**<0.01**
Femoral neck	20/59 (34)	7/31 (23)	13/28 (46)	0.05
Prevalence of Osteoporosis (*n*_*c*_/*n*_*t*_ (%))				
Whole-body	5/62 (8)	1/32 (3)	4/30 (13)	0.14
Lumbar spine	5/60 (8)	1/33 (3)	4/27 (15)	0.10
Total hip	10/67 (15)	2/34 (6)	8/33 (24)	**0.04**
Femoral neck	7/59 (12)	1/31 (3)	6/28 (21)	**0.03**

Normally distributed data are presented as mean ± SD, while non-normally distributed data (*) are presented as median (Q1-Q3). Low bone mineral density is defined as bone mineral density Z score < −1.0; Osteoporosis is defined as bone mineral density *Z*-score < −2.0. *n*_c_ = number of athletes with low bone mineral density or osteoporosis. *n*_t_ = total number of analyzed scans. Differences between wheelchair-dependent and non-wheelchair-dependent athletes in absolute bone mineral density and bone mineral density *Z*-scores are analyzed with a student t-test. Statistical differences in prevalence of low bone mineral density and osteoporosis are analyzed with a χ^2^-test. Statistical significance (P < 0.05) is indicated in bold.

### 3.2 Bone mineral density

A total of 8, 10, 3 and 11 scans of the whole-body, lumbar spine, total hip, and femoral neck, respectively, had to be excluded due to the presence of metal implants or deformations of the spine or hip. Additionally, the right instead of the left hip was analyzed for 6 participants because of deformations or metal in the left hip.

**Fig. 1:**
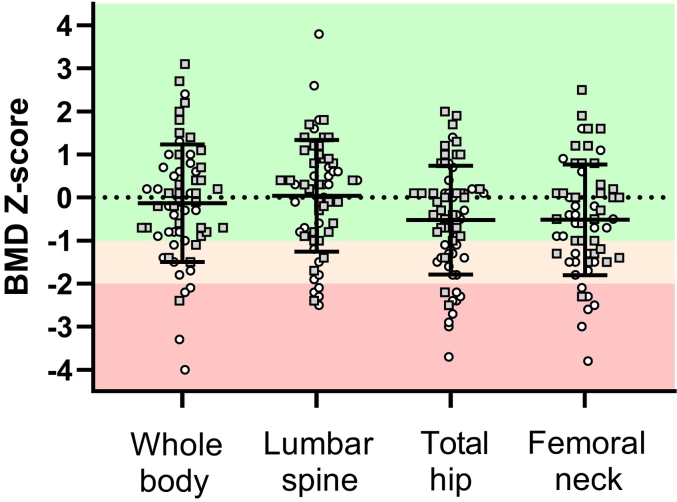
Bone mineral density *Z*-scores in all Paralympic athletes at different measurement sites. Data are presented as individual scores, while the horizontal bars with error bars represent means±SD. Gray squares = Non-wheelchair-dependent athlete; white circles = wheelchair-dependent athlete; BMD = bone mineral density.

Across all participants, the mean BMD *Z*-scores were -0.1 ± 1.4, 0.0 ± 1.3, −0.5 ± 1.3 and -0.5 ± 1.3 at the whole-body, lumbar spine, total hip, and femoral neck respectively (Fig. 1 and Table 2). The prevalence of low BMD (*Z*-score < −1.0) was highest at the femoral neck (34 %), followed by the total hip (31 %), whole-body (21 %) and lumbar spine (18 %). The prevalence of osteoporosis (*Z*-score < −2.0) was highest at the total hip (15 %), followed by the femoral neck (12 %), lumbar spine (8 %), and whole-body (8 %).

**Fig. 2:**
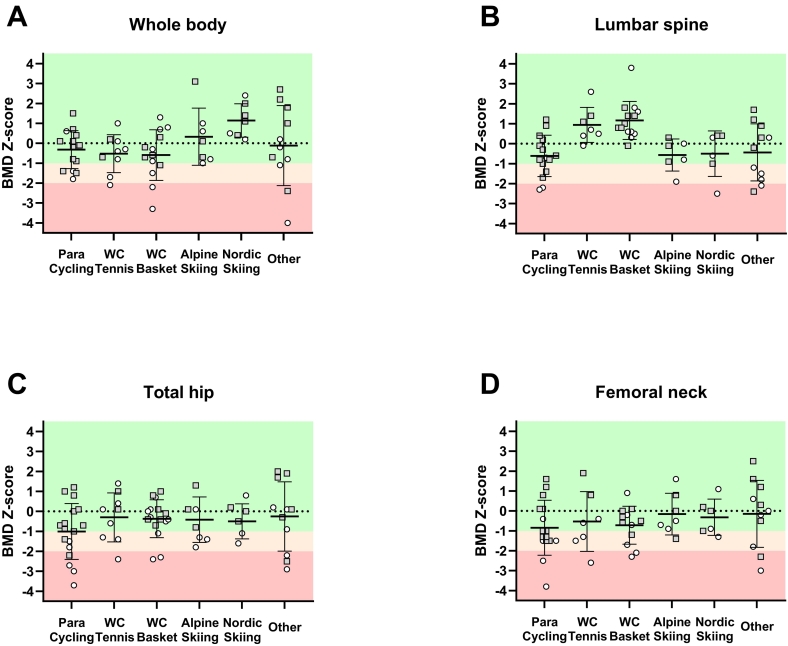
The bone mineral density Z-scores at whole-body level (A), lumbar spine (B), total hip (C) and femoral neck (D) for the various sports. At the lumbar spine (B) there is a significant difference in BMD Z-scores between wheelchair tennis players and Para cyclists and between wheelchair basketball players and Para cyclists, alpine skiers, Nordic skiers, and ‘other’ athletes; *P* < 0.05 for all comparisons. Gray squares = Non-wheelchair-dependent athlete; white circles = wheelchair-dependent athlete; BMD = bone mineral density. WC = wheelchair; Basket = basketball.

When examining the sports categories separately (Table 3), the prevalence of low BMD at the whole-body level (Fig. 2A) was absent for the Para alpine skiers and Para Nordic skiers, with mean BMD *Z*-scores well above zero (0.3 ± 1.4 and 1.1 ± 0.8, respectively). The mean whole-body BMD Z-scores for Para cycling, wheelchair tennis, wheelchair basketball and ‘other sports’ were -0.3 ± 1.0, −0.5 ± 1.0, −0.6 ± 1.3 and -0.1 ± 2.0, respectively. No significant differences between the BMD *Z*-scores of the whole-body were found between sports.

**Table 3:** Bone mineral density of the Paralympic athletes from various sports at different skeletal sites.

	Total	Para cycling	Wheelchair tennis	Wheelchair Basketball	Para alpine skiing	Para Nordic skiing	Other
Whole-body (n) BMD (g/cm^2^) BMD Z-score Low BMD (%) Osteoporosis (%)	62 1.134 ± 0.122 −0.1 ± 1.4 21 8	15 1.118 ± 0.127 −0.3 ± 1.0 27 0	9 1.126 ± 0.070 −0.5 ± 1.0 22 11	13 1.077 ± 0.105 −0.6 ± 1.3 31 15	7 1.187 ± 0.132 0.3 ± 1.4 0 0	7 1.204 ± 0.076 1.1 ± 0.8 0 0	11 1.149 ± 0.165 −0.1 ± 2.0 27 18
Lumbar spine (n) BMD (g/cm^2^) sBMD (g/cm^2^) BMD Z-score Low BMD (%) Osteoporosis (%)	59 1.083 ± 0.158 1.130 ± 0.162 0.0 ± 1.3 18 8	15 0.989 ± 0.128 1.055 ± 0.148 −0.6 ± 1.0 27 13	7 1.163 ± 0.113 1.245 ± 0.120 0.9 ± 0.9^a^ 0 0	14 1.168 ± 0.099^a^ 1.252 ± 0.102^a^ 1.2 ± 1.0^a^ 0 0	6 1.021 ± 0.146 1.070 ± 0.106 −0.6 ± 0.8^b^ 17 0	6 1.127 ± 0.169 1.070 ± 0.163 −0.5 ± 1.1^b^ 17 17	11 1.080 ± 0.206 1.068 ± 0.180^b^ −0.4 ± 1.4^b^ 46 18
Total hip (n) BMD (g/cm^2^) sBMD (g/cm^2^) BMD Z-score Low BMD (%) Osteoporosis (%)	66 0.916 ± 0.187 0.909 ± 0.183 −0.5 ± 1.3 31 15	17 0.844 ± 0.212 0.843 ± 0.220 −1.0 ± 1.4 41 24	9 0.942 ± 0.171 0.956 ± 0.172 −0.3 ± 1.2 33 11	16 0.913 ± 0.138 0.921 ± 0.134 −0.4 ± 1.0 19 13	7 0.894 ± 0.139 0.897 ± 0.116 −0.5 ± 1.1 43 0	6 0.980 ± 0.134 0.921 ± 0.147 −0.4 ± 0.9 29 0	11 0.989 ± 0.255 0.959 ± 0.239 −0.3 ± 1.7 27 27
Femoral neck (n) BMD (g/cm^2^) sBMD (g/cm^2^) BMD Z-score Low BMD (%) Osteoporosis (%)	59 0.845 ± 0.199 0.882 ± 0.186 −0.5 ± 1.3 34 12	16 0.772 ± 0.189 0.832 ± 0.226 −0.8 ± 1.4 50 13	7 0.813 ± 0.198 0.903 ± 0.215 −0.5 ± 1.5 43 14	12 0.769 ± 0.105 0.855 ± 0.114 −0.7 ± 0.9 33 17	7 0.870 ± 0.151 0.935 ± 0.109 −0.2 ± 1.0 14 0	6 0.984 ± 0.138 0.886 ± 0.158 −0.3 ± 0.9 17 0	11 0.964 ± 0.274 0.937 ± 0.226 −0.1 ± 1.7 27 18

Bone mineral density *Z*-scores are presented as mean ± SD. BMD = bone mineral density; sBMD = standardized bone mineral density; low BMD is defined as a Z-score < −1; osteoporosis is defined as Z-score < −2. sBMD is calculated based on the equations by Hui et al. *(*Hui et al., *1997**)* (for the lumbar spine) and by Lu et al. *(*Lu et al., *2001**)* (for the total hip and femoral neck).

a Significantly different from Para cycling *P* < 0.05.

b Significantly different from wheelchair basketball *P* < 0.05.

At the lumbar spine (Fig. 2B), low BMD was absent in wheelchair tennis and wheelchair basketball athletes. Mean BMD *Z*-scores were significantly higher in wheelchair basketball players (1.2 ± 1.0) compared with Para cycling (−0.6 ± 1.0), Para alpine skiing (−0.6 ± 0.8), Para Nordic skiing (−0.5 ± 1.1), and ‘other sports’ (−0.4 ± 1.4; *P* < 0.05 for all comparisons). Wheelchair tennis players also had a significantly higher BMD *Z*-scores at the lumbar spine (0.9 ± 0.9) compared with the Para cyclists (*P* = 0.04).

At the total hip (Fig. 2C), the mean BMD Z-scores were − 1.0 ± 1.4, −0.3 ± 1.2, −0.4 ± 1.0, −0.5 ± 1.1, −0.4 ± 0.9 and − 0.3 ± 1.7 for the para-cyclists, wheelchair tennis, wheelchair basketball, alpine skiers, Nordic skiers, and ‘other sports’ athletes respectively. No significant differences in mean BMD *Z*-scores were observed between sports.

At the femoral neck (Fig. 2D), the mean BMD *Z*-scores were − 0.8 ± 1.4, −0.5 ± 1.5, −0.7 ± 0.9, −0.2 ± 1.0, −0.3 ± 0.9 and − 0.1 ± 1.7 for the para-cyclists, wheelchair tennis, wheelchair basketball, alpine skiers, Nordic skiers, and ‘other sports’ athletes respectively. For this skeletal site, also no significant differences in mean BMD Z-scores were observed between sports.

When comparing BMD *Z*-scores between wheelchair-dependent and non-wheelchair-dependent athletes, wheelchair-dependent athletes appeared to have significantly lower BMD *Z*-scores at the whole-body (−0.5 ± 1.4 vs 0.2 ± 1.3; *P* = 0.04), total hip (−1.1 ± 1.2 vs 0.0 ± 1.1; *P* < 0.01) and femoral neck (−1.0 ± 1.3 vs −0.1 ± 1.2; *P* < 0.01), but not at the lumbar spine (−0.1 ± 1.6 vs 0.2 ± 1.0; *P* = 0.20) compared to the non-wheelchair-dependent athletes (Table 2).

### 3.3 Regression analyses

As shown in Table 4, the final regression model did not reach statistical significance for whole-body BMD *Z*-score, *F*(1,55) = 4.009, *P* = 0.05, adjusted *R*^2^ = 0.05.

**Table 4:** Backward stepwise multiple regression model with whole-body, lumbar spine, total hip and femoral neck bone mineral density Z-scores as dependent variables. Statistical significance (P < 0.05) is indicated in bold.

Outcome variable: Z-score whole-body	Unstandardized Coefficients		Standardized Coefficients			
	B	Std. Error	Beta (β)	t	*R*^*2*^_*adj*_	*P*-value
Model					0.051	0.050
(constant)	0.279	0.252		1.110		0.272
Wheelchair dependence	−0.719	0.359	−0.261	−2.002		0.050


Outcome variable: Z-score lumbar spine	Unstandardized Coefficients		Standardized Coefficients			
	B	Std. Error	Beta (β)	t	*R*^*2*^_*adj*_	*P*-value
Model					0.431	**<0.001**
(constant)	−2.274	0.660		−3.447		**0.001**
Body mass	0.027	0.009	0.293	2.820		**0.007**
Wheelchair tennis	1.497	0.436	0.363	3.431		**0.001**
Wheelchair basketball	1.684	0.321	0.555	5.241		**<0.001**


Outcome variable: Z-score Total hip	Unstandardized Coefficients		Standardized Coefficients			
	B	Std. Error	Beta (β)	t	*R*^*2*^_*adj*_	*P*-value
Model					0.337	**<0.001**
(constant)	−1.473	0.824		−1.789		0.079
Body mass	0.026	0.011	0.274	2.367		**0.021**
Wheelchair dependence	−1.048	0.306	−0.404	−3.422		**0.001**
Para cycling	−0.927	0.324	−0.307	−2.859		**0.006**


Outcome variable: Z-score Femoral Neck	Unstandardized Coefficients		Standardized Coefficients			
	B	Std. Error	Beta (β)	t	*R*^*2*^_*adj*_	*P*-value
Model					0.200	**0.001**
(constant)	−1.957	0.967		−2.025		**0.048**
Body mass	0.028	0.013	0.285	2.108		**0.040**
Wheelchair dependence	−0.736	0.355	−0.280	−2.073		**0.043**

For the lumbar spine BMD Z-score, the final regression model (Table 4) contained body mass, wheelchair tennis and wheelchair basketball as factors positively associated with BMD, *F*(3, 50) = 14.372, *P* < 0.001, adjusted *R*^2^ = 0.43.

For the total hip BMD Z-score, the final regression model (Table 4) contained body mass as factor positively associated with BMD, while wheelchair dependence and Para cycling were negatively associated with BMD, *F*(3, 57) = 11.165, *P* < 0.001, adjusted *R*^2^ = 0.34.

For the femoral neck BMD Z-score, the final regression model (Table 4) contained body mass as factor positively associated with BMD, while wheelchair dependence was negatively associated with BMD, *F*(2, 52) = 7.741, *P* = 0.001, adjusted *R*^2^ = 0.20.

## 4 Discussion

The current study revealed that low BMD in Paralympic athletes is mainly prevalent in the hip region. More specifically, BMD of the hip region seemed to be particularly affected in wheelchair-dependent athletes. In contrast, the overall prevalence of low BMD at both the lumbar spine and whole-body level was not notably high. Wheelchair basketball and tennis players even seemed to be fully protected against low BMD of the lumbar spine. Regression analyses identified the type of sport, wheelchair dependence, and body mass as the main predictors of BMD in Paralympic athletes.

### 4.1 Prevalence of low bone mineral density

When considering *Z*-scores of a normal distribution curve, approximately 16 % of the investigated population can be expected to have a low BMD (Z-score < −1). In this regard, the prevalence of low BMD in Paralympic athletes was quite substantial at the total hip (31 %) and femoral neck (34 %), while the prevalence of low BMD was not notably high at the whole-body level (21 %) and lumbar spine (18 %). In contrast to our cohort of Paralympic athletes, it has previously been shown that non-athlete individuals with a disability have a considerably higher prevalence of low BMD at the lumbar spine (31 %) and at the hip (42 %) (Smith et al., 2009). This supports the notion that sport participation generally exerts a positive effect on BMD in people with a disability (Blauwet et al., 2019). Moreover, previous studies have shown that wheelchair-dependent athletes had a slightly, but sometimes significantly, higher BMD at the spine compared to a non-athlete reference population (Bauman et al., 1999; Sutton et al., 2009). Nevertheless, the Paralympic athletes in our study generally seem to display lower mean BMD *Z*-scores (Morel et al., 2001) and a higher prevalence of low BMD Z-scores (Tenforde et al., 2018) compared with non-disabled athletes.

### 4.2 The importance of mechanical loading

The high prevalence of low BMD in the hip region can be mainly attributed to the wheelchair-dependent athletes. Indeed, the wheelchair-dependent athletes had a significantly lower BMD at the total hip and femoral neck compared to the non-wheelchair-dependent athletes. This finding was confirmed by the regression analyses, and is consistent with previous research showing that wheelchair athletes have a lower BMD in the legs compared to athletes (Miyahara et al., 2008) and non-athletes (Sutton et al., 2009) without a disability. The high prevalence of low BMD among wheelchair-dependent athletes can probably be explained by the low mechanical loading on the lower body. In this regard, the lack of local mechanical loading prevents proper bone stress and subsequent bone remodeling at specific skeletal sites (Robling et al., 2006). Mechanical loading is not only affected by wheelchair use, but also by body mass. A higher body mass leads to more mechanical stress on the bones, due to higher gravitational forces, which leads to a higher BMD (Felson et al., 1993). The relationship between body mass or body mass index and BMD has been well established in the elderly population (Felson et al., 1993; Broussard and Magnus, 2004). This positive relationship is also confirmed by the regression analyses in the current study, which identified body mass as a predictor of BMD at the lumbar spine, total hip, and femoral neck, independent of wheelchair dependence.

### 4.3 Differences in bone mineral density between sports

Importantly, the differences in loading patterns between sports, seem to explain a large part of the differences in BMD between sports. Para cycling was identified as an independent risk factor for low BMD at the total hip and femoral neck. Such a low BMD of the hip and femoral neck is also seen in elite road-race cyclists without a disability (Hilkens et al., 2023), although in that population low BMD was even more pronounced at the lumbar spine. Consequently, Para cyclists and wheelchair-dependent athletes should be aware of their increased risk of low BMD of the hip region. Therefore, preventive and treatment strategies for bone health should also target the hip region, acknowledging that this might be challenging in wheelchair-dependent individuals. Wheelchair tennis and basketball players exhibited relatively high BMD *Z*-scores at the lumbar spine, and none of these athletes had a BMD Z-score < −1. These findings suggest that, despite the majority of wheelchair tennis and basketball athletes being wheelchair-dependent (78 % and 61 %, respectively), the lumbar spine remains protected against low BMD. Apparently, athletes in these particular sports receive highly effective osteogenic stimuli for the lumbar spine. When examining the biomechanics of wheelchair tennis and basketball, it is obvious that these sports involve a significant number of rotational movements that could affect the BMD by generating torsional loading. Previous research has indicated that torsional loading creates the greatest strain upon bone compared with other loading directions (Rubin et al., 1996). Additionally, studies on non-disabled tennis and baseball players have demonstrated that torsional loading on the racket and throwing arm, respectively, account for the biggest changes in BMD (Ireland et al., 2013; Warden et al., 2009). As a result, the high levels of torsional loading on the lumbar spine in wheelchair basketball and tennis may explain the differences in BMD *Z*-scores and the low prevalence of low BMD of the lumbar spine compared with the other Paralympic sports. These results also illustrate the principle of site-specific loading, since the relatively high BMD of the lumbar spine in these particular sports was not seen at the whole-body level or hip region. It could be suggested that torsional loading should be incorporated within the training programs of Paralympic athletes as a potential protective measure against low BMD of the lumbar spine. Furthermore, to decrease fracture rates it would be interesting to use torsional loading on other skeletal sites with low BMD, where possible.

### 4.4 Other potential risk factors for low bone mineral density

A known risk factor for low BMD is a low energy availability (Mountjoy et al., 2018). Indeed, we have previously identified serum T3, a marker of low energy availability (ACSM, 2007; Loucks and Heath, 1994), as predictor of low BMD in elite cyclists (Hilkens et al., 2023). In the current study, however, serum T3 concentrations were not associated with BMD. It can be speculated that the heterogenous loading patterns and low mechanical loading due to wheelchair usage and the various sports, may have masked the impact of energy availability on BMD in this population. This argument may also explain why serum vitamin D was not associated with BMD. Moreover, it should be noted that most athletes in our study used vitamin D or multivitamin supplements, and 83 % of the athletes had a vitamin D level exceeding 50 nmol/L. Such vitamin D levels may be adequate to support optimal bone health (Del Valle et al., 2011). Besides low energy availability and vitamin D status, also the duration of disability has previously been linked to bone health (Clasey et al., 2004). However, we found no association between the duration of disability and BMD. This could be due to the relatively long time since disability onset, averaging 21 years. For example, demineralization of the proximal femur starts within the first 6 months after spinal cord injury, stabilizing at 60–70 % of normal values after 12 to 24 months (Biering-Sørensen et al., 1990; Jiang et al., 2006). Therefore, it seems likely that the loss of BMD following disability onset has stabilized for most athletes in our study, making it a non-discriminatory risk factor in this cohort. It is also worth noting that 51 % of the athletes were congenitally disabled, which could have influenced the potential relationship between time since disability and the BMD.

### 4.5 Strengths and limitations

The strength of the current study is the comprehensive assessment of BMD and associated risk factors in a representative population of Paralympic athletes, covering a large variety of Paralympic sport disciplines and disabilities. Nevertheless, we should also acknowledge some limitations. Inherent to the Paralympic population and variety of sport disciplines, the current cohort was highly heterogeneous in terms of disabilities and loading patterns. This heterogeneity in loading patterns may have limited the statistical power to identify risk factors for low BMD other than loading-related factors. Secondly, due to the international nature of the study, BMD was assessed at two different research sites using three different DXA systems from two manufacturers. Although this might increase variability in DXA outcomes, operating procedures were standardized across research sites, thereby minimizing potential measurement errors. While absolute BMD values may slightly vary between DXA systems from different manufacturers (Hui et al., 1997; Lu et al., 2001; Shepherd et al., 2012) each manufacturer employs its own reference database, making differences in *Z*-scores between systems likely negligible. Moreover, to account for potential discrepancies in absolute BMD values across different DXA systems, we have also reported standardized BMD values. A third limitation is that it remains to be established whether low BMD Z-scores in Paralympic athletes are also related to increased risk for traumatic fractures or bone stress injuries. Finally, 34 individual scans had to be excluded from the analysis due to deformities or metal implants. It can be argued that athletes with a deformed hip or lumbar spine receive less mechanical loading at these sites, potentially leading to lower BMD. Consequently, the results reported in this study may underestimate the true prevalence of low BMD in Paralympic athletes.

## 5 Conclusions

In this cohort of Paralympic athletes, low BMD is highly prevalent in the hip region, but not necessarily at the lumbar spine. Wheelchair dependence, body mass, and type of sport are the main factors associated with BMD in this population. The relatively high BMD of the lumbar spine in wheelchair tennis and basketball suggests that the typical torsional loadings patterns in these sports may be protective against low BMD of the lumbar spine. The implementation of such torsional loading patterns may warrant consideration in other Paralympic disciplines, as well as in skeletal sites that are susceptible to bone stress injuries or traumatic fractures.

The following is the supplementary data related to this article.

Table S1 Reference values for P1NP and CTX according to age and sex. Table S1

## CRediT authorship contribution statement

**Vera C.R. Weijer:** Writing – original draft, Visualization, Methodology, Investigation, Formal analysis, Data curation, Conceptualization. **Jan-Willem van Dijk:** Writing – review & editing, Supervision, Project administration, Methodology, Investigation, Funding acquisition, Conceptualization. **Lotte van Dam:** Writing – review & editing, Investigation, Data curation. **Linn Risvang:** Writing – review & editing, Methodology, Investigation, Formal analysis, Data curation. **Judith Bons:** Writing – review & editing, Validation, Methodology, Formal analysis. **Truls Raastad:** Writing – review & editing, Project administration, Funding acquisition, Conceptualization. **Luc J.C. van Loon:** Writing – review & editing, Supervision, Funding acquisition, Conceptualization. **Kristin L. Jonvik:** Writing – review & editing, Methodology, Data curation, Conceptualization.

## Declaration of competing interest

No conflicts of interest, financial or otherwise, are declared by the authors. The results of this study are presented clearly, honestly, and without fabrication, falsification, or inappropriate data manipulation. Results of this study were previously presented in poster format at ISENC (international sport and exercise nutrition conference) and additionally an abstract was published in IJSNEM (International Journal of Sport Nutrition and Exercise Metabolism).

## Data Availability

Data will be made available on request.
