# Supplementary material for: Do Paralympic athletes suffer from brittle bones? Prevalence and risk factors of low bone mineral density in Paralympic athletes
Source: Bone Rep. 2024 Apr 18;21:101767. doi: 10.1016/j.bonr.2024.101767 (PMC11061701; doi:10.1016/j.bonr.2024.101767)
Supplement: Table S1 — Reference values for P1NP and CTX according to age and sex. [file mmc1.docx]

**SUPPLEMENTAL**

**Table S1.** Reference values for P1NP and CTX according to age and sex.

| **Sex** | **Age (years)** | **P1NP (ng/mL)** | | **Age (years)** | **CTX (ng/mL)** | |
| --- | --- | --- | --- | --- | --- | --- |
|  |  | **Lower limit** | **Upper limit** |  | **Lower limit** | **Upper limit** |
| **Women** | 17-20 | 25.2 | 160 | 14-18 | 0.32 | 2.62 |
|  | 21-35 | 14.7 | 74.6 | >18 & premenopausal | 0.05 | 0.67 |
|  | 35-50 | 12.9 | 66.8 |  |  |  |
| **Men** | 17-20 | 28.1 | 369 | 14-18 | 1.02 | 4.47 |
|  | 21-45 | 19.4 | 95.4 | 25-29 | 0.12 | 0.83 |
|  | >45 | 12.8 | 71.9 | 30-34 | 0.11 | 0.81 |
|  |  |  |  | 35-39 | 0.11 | 0.78 |
|  |  |  |  | 40-44 | 0.10 | 0.76 |
|  |  |  |  | 45-49 | 0.09 | 0.73 |

P1NP reference values are based on the study of Morovat *et al.* (1). CTX reference values are based on the study of Michelsen *et al* (2) and Diemar *et al*. (3).

**References:**

1. Morovat A, Catchpole A, Meurisse A, Carlisi A, Bekaert A-C, Rousselle O, et al. IDS iSYS automated intact procollagen-1-N-terminus pro-peptide assay: method evaluation and reference intervals in adults and children. Clinical Chemistry and Laboratory Medicine (CCLM). 2013;51(10):2009-18.

2. Michelsen J, Wallaschofski H, Friedrich N, Spielhagen C, Rettig R, Ittermann T, et al. Reference intervals for serum concentrations of three bone turnover markers for men and women. Bone. 2013;57(2):399-404.

3. Diemar SS, Lylloff L, Rønne MS, Møllehave LT, Heidemann M, Thuesen BH, et al. Reference intervals in Danish children and adolescents for bone turnover markers carboxy-terminal cross-linked telopeptide of type I collagen (β-CTX), pro-collagen type I N-terminal propeptide (PINP), osteocalcin (OC) and bone-specific alkaline phosphatase (bone ALP). Bone. 2021;146:115879.
